# Effectiveness of Play Therapy in Reducing Anxiety During the Preoperative Period in Pediatric Patients Undergoing Inguinal Hernioplasty

**DOI:** 10.7759/cureus.101160

**Published:** 2026-01-09

**Authors:** Carlos Felipe Rodríguez-González, Lilia Esther Ramírez-Plascencia, Sara Paulina Miranda-Brambila, Sonia Sifuentes-Franco, Daniel Arellano Silva, Erika Martínez-López, Juan José Rivera-Valdés

**Affiliations:** 1 Anesthesiology Service, Civil Hospital of Guadalajara Dr. Juan I. Menchaca, Guadalajara, MEX; 2 Clinical Science Laboratory, Department of Health Sciences, Los Valles Campus, University of Guadalajara, Ameca, MEX; 3 Department of Anesthesiology, Jalisco Institute of Cancerology, Zapopan, MEX; 4 Department of Molecular Biology and Genomics, University Center of Health Sciences, Institute of Translational Nutrigenetics and Nutrigenomics, University of Guadalajara, Guadalajara, MEX

**Keywords:** anxiety, play therapy, preoperative care, surgery, visual analog scale

## Abstract

Introduction: The preoperative period is challenging in children due to increased anxiety. Preoperative anxiety results in increased postoperative pain, insomnia, nausea, fatigue, and reduced effectiveness of anesthesia and analgesia. Therefore, this study aimed to compare the efficacy of a play therapy intervention versus standard pharmacological premedication in reducing preoperative anxiety in pediatric patients undergoing inguinal hernioplasty.

Methods: A randomized controlled trial was conducted at Dr. Juan I. Menchaca Civil Hospital, Guadalajara, Mexico, involving 32 pediatric patients aged 3 to 6 years undergoing inguinal hernioplasty. Patients were randomized into two groups: (1) an intervention group, which received play therapy involving a transport cart and toys, and (2) a control group, which received anxiolytic medication and standard care. Patient anxiety was assessed using the modified Yale Preoperative Anxiety Scale-short form (mYPAS-SF), as a primary outcome, and the Visual Analogue Anxiety Scale for Parents (VAS-P), as a secondary outcome, at three different times prior to surgery: initial contact, post-intervention, and transfer to the preoperative area.

Results: VAS-P only showed significant differences at the final evaluation point, with lower anxiety scores observed in the control group. mYPAS showed significant differences in the score given at the post-intervention evaluation and in the number of patients considered anxious (>30 points), both of which were lower in the play therapy group. At the final evaluation, the number of patients considered anxious was lower in the play therapy group.

Conclusion: Play therapy proves to be an effective and safe intervention for reducing anxiety in children during the preoperative period. Its incorporation into standard clinical practices for pediatric surgery could enhance emotional management and improve surgical outcomes.

## Introduction

The perioperative period presents significant challenges for pediatric patients. According to a review that included 12 studies that reported preoperative anxiety in children, it showed an incidence of 41.7% to 75.44% [1]. Anxiety is a common concern during the preoperative phase, often leading to functional dysregulation in the central nervous system through changes in neural circuits, neurotransmitters, and key neuroendocrine systems that normally maintain emotional balance and homeostasis such as hypothalamic-pituitary-adrenal (HPA) axis [2-4]. These alterations are linked to increased postoperative pain, along with complications such as insomnia, nausea, fatigue, and reduced effectiveness of anesthesia and analgesia [5-7]. These complications not only affect patient well-being but also increase the duration of stay in the perioperative holding area or post anesthetic care units, increasing the duration of stay in hospital which significantly increase healthcare and family costs.

Several factors have been identified as predictors of preoperative anxiety, including sex, age, cognitive ability, baseline anxiety, temperament, prior medical experiences, and parental anxiety. These variables should be carefully considered when developing interventions to improve emotional management in pediatric patients [1,8]. Therefore, implementing effective strategies to mitigate anxiety is crucial in pediatric surgical care.

Play therapy has been used for decades as an effective strategy to prepare pediatric patients for invasive medical procedures. This intervention has been recognized for providing multiple benefits, including reducing anxiety and postoperative pain in hospitalized children [9]. Developed in the late 20th century, play therapy encompasses a variety of treatment methods that harness the therapeutic benefits of play. It is defined as a method that allows children to express their emotions and experiences in a supportive environment, utilizing play as their natural medium for self-expression. Through play, the child can release pent-up feelings such as tension, frustration, insecurity, fear, and confusion [10]. Therefore, the primary objective of this randomized controlled trial was to compare the efficacy of a play therapy intervention versus standard pharmacological premedication, oral midazolam, in reducing preoperative anxiety in pediatric patients undergoing inguinal hernioplasty.

## Materials and methods

Design and study population

A randomized, controlled clinical trial with parallel groups was conducted on pediatric patients who were scheduled for inguinal hernioplasty at the Dr. Juan I. Menchaca Civil Hospital in Guadalajara, Mexico, from January 2023 to July 2023. As inclusion criteria, we considered pediatric patients aged 3 to 6 years, of any sex, scheduled for elective inguinal hernioplasty, undergoing their first anesthetic-surgical procedure, and whose legal guardian provided informed consent. Exclusion criteria included patients with known allergies to midazolam or with abnormal perinatal history (e.g., neonatal hypoxia or chromosomal abnormalities), as well as patients undergoing emergency inguinal hernioplasty due to complications such as strangulation or incarceration. Once the parents' informed consent was obtained, participants were divided into two groups: the play therapy and the control group.

Sample size

The sample size calculation was carried out using the formula for the difference of means, expecting an average difference in the preoperative anxiety score of 10 points between groups, with a 95% confidence interval, 80% power, and a variance of 100. A total of 32 patients were obtained, 16 per group.

Randomization and allocation

The simple randomization method was used, utilizing a previously generated table of random numbers. Random assignment was implemented using opaque, sealed, and consecutively numbered envelopes. Each envelope contained the indication of the group and the treatment assigned according to the previously established random sequence. This was done at the first contact with the anesthesiologist on the day of the elective procedure.

Study groups

The play therapy included pediatric patients, who received play therapy involving toys. In the Pediatric Anesthesia Room, the play was free and provided by a medical resident from the anesthesiology service, and his role was observational, making sure the child's safety was not compromised while playing. After playing with a transport cart for five minutes, they were transported to the operating room using the same cart. The control group included patients of the same age, who received oral midazolam at a dose of 0.1 mg/kg in the same room by the anesthesiologist, and after five minutes, they were transferred to the operating room either in caregiver's arms or on a standard stretcher. This study was performed in line with the principles of the Declaration of Helsinki. It was approved by the Ethics and Research Committees (CONBIOETICA-14-CEI-008-20161212 on January 19, 2023) and was registered at clinical trials (NCT06868420).

Assessment and management of anxiety

As the primary outcome, the patient’s anxiety levels were assessed by the anesthesiologist using the modified Yale Preoperative Anxiety Scale-Short Form (mYPAS-SF) [11], which provides scores ranging from 22.9 to 100 points, with values greater than 30 indicating the presence of significant anxiety. Besides, the child's anxiety levels were assessed by their parent or legal guardian, as a secondary outcome, using the Visual Analog Anxiety Scale for Parents (VAS-P), which is a 10 cm horizontal scale, where values greater than 5 cm indicate significant anxiety. Consequently, the proportion of anxious patients was calculated as a secondary outcome. Both instruments were used at three time points: upon first contact with the anesthesiologist, after the intervention (play-based or pharmacological), and upon arrival in the operating room.

Statistical analysis

Descriptive statistics were used to present the main clinical and demographic characteristics, including measures of central tendency and dispersion. The Mann-Whitney U test was applied to identify differences between the study groups in quantitative variables, while the chi-square (χ²) test was used for categorical variables. A p-value of <0.05 was considered statistically significant. All analyses were conducted using IBM SPSS Statistics for Windows, Version 27.0 (Released 2020; IBM Corp., Armonk, New York, United States) and R Statistical Software. 

## Results

**Figure 1:**
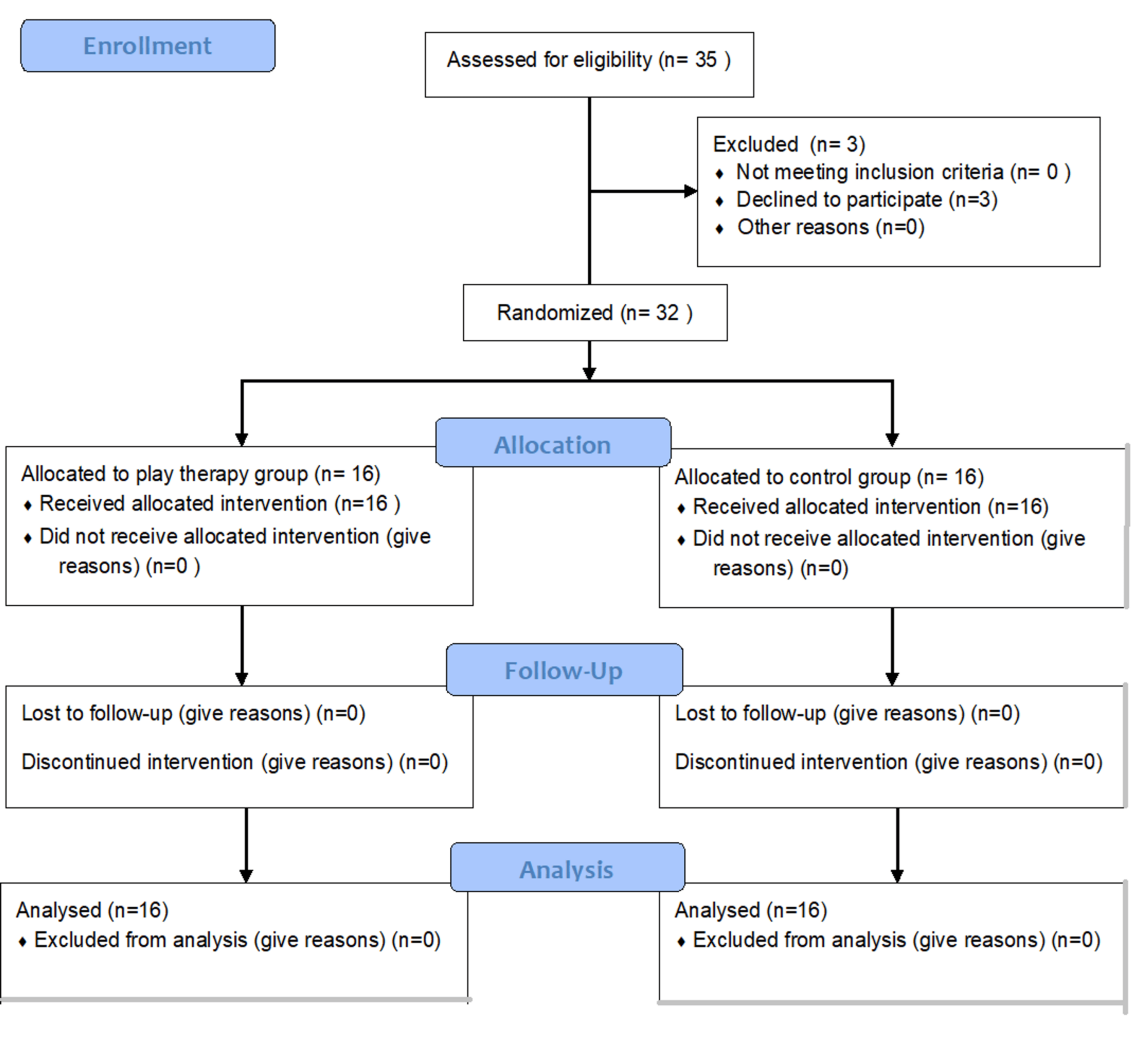
CONSORT flow diagram. Source: Adapted from CONSORT 2010 flow diagram [12].

A total of 32 pediatric patients aged 3 to 6 years were included in the study, comprising 27 males (84.4%) and five females (15.6%) (Figure 1). The type of hernia was identified prior to the study for each patient: 63.1% (n=57) had a direct hernia, while 46.9% (n=15) had an indirect hernia. Additionally, the corresponding American Society of Anesthesiologists (ASA) classification was determined, with 27 patients (84.1%) classified as Grade I and the remaining five patients (15.6%) as Grade II. Among these, only two patients (6.3%) from the play therapy group presented comorbidities at the time of surgery (Table 1). No complications were reported in any patients during the perioperative period.

**Table 1:** Demographic characteristics. ASA: American Society of Anesthesiologists.

Characteristic	Control group (n=16)	Play therapy group (n=16)	p
Gender			0.500
Male, n (%)	14 (87.5)	13 (81.2)	
Female, n (%)	2 (12.5)	3 (18.8)	
Age, years	4.4 ± 1.2	4.7 ± 1.01	0.533
Inguinal hernia classification, n (%)			0.240
Direct	10 (62.5)	7 (43.8)	
Indirect	6 (37.5)	9 (56.3)	
ASA, n (%)			0.500
ASA I	14 (87.5)	13 (81.2)	
ASA II	2 (12.5)	3 (18.8)	

The scores from the VAS-P and mYPAS-SF scales were recorded at three different time points: at the initial contact with the evaluator, after the pharmacological or play-based intervention, and during the transfer to the preoperative holding area (gray area). The mean, minimum, and maximum scores for each group are presented in Table 2.

**Table 2:** Scores by group on the VAS-P and mYPAS-SF scales at the three preoperative evaluation moments. mYPAS-SF: modified Yale Preoperative Anxiety Scale-Short Form; VAS-P: Visual Analog Anxiety Scale for Parents. *p < 0.05. **p < 0.01.

	Control group N (%) or mean ± SD	Play therapy group N (%) or mean ± SD	p
VAS-P score (first contact)			
Average	4.8±2.7	3.7±2.5	0.094
Minimum	0	1	
Maximum	8	10	
Anxiety (VAS-P≥5)	10 (62.5)	5 (31.3)	0.077
VAS-P score (post-intervention)			
Average	2.2±2.3	2.3±1.3	0.072
Minimum	0	0	
Maximum	7	5	
Anxiety (VAS-P≥5)	3 (18.8)	2 (12.5)	0.626
VAS score (gray area)			
Average	1.7±1.9	3.1±2.2	0.036*
Minimum	0	0	
Maximum	6	8	
Anxiety (VAS-P≥5)	2 (12.5)	3 (18.8)	0.626
mYPAS score (first contact)			
Average	48.1±14.8	28.9±6.3	0.350
Minimum	27	23	
Maximum	75	45	
Anxiety (mYPAS>30)	14 (87.5)	7 (43.8)	0.009**
mYPAS score (post-intervention)			
Average	33±9.1	25.3±3.0	0.004**
Minimum	23	23	
Maximum	46	35	
Anxiety (mYPAS>30)	8 (50)	1 (6.3)	0.006**
mYPAS score (gray area)			
Average	29.4±8.9	26.9±4.9	0.442
Minimum	23	23	
Maximum	54	39	
Anxiety (mYPAS>30)	5 (31.3)	2 (12.5)	0.200

## Discussion

Since perioperative anxiety is a major concern among patients undergoing surgery due to its association with an increased risk of complications such as night terrors, nocturnal enuresis, and feeding difficulties [8], this study aimed to analyze the effectiveness of non-pharmacological strategies in pediatric patients, specifically the use of play and transportation in a toy car during the preoperative period.

In this study, we chose to work with elective inguinal hernia surgery in patients aged 3 to 6 years because it is the most common elective surgery in the pediatric population in our institution. Among a total of 32 patients, there were no significant differences in the age or sex distribution between the groups. Additionally, there was no significant difference in the types of hernias found, and no complications were reported in either group.

It is important to emphasize that two instruments were used to assess the anxiety levels of the pediatric patients. Parents assessed their children's anxiety using a 10-cm VAS-P [13], while the anesthesiologist used the mYPAS scale. Although both instruments have been widely used in different clinical studies, they are generally applied independently [13-15].

Three time points were selected to measure the patient's anxiety levels in the preoperative period: initial contact with the anesthesiologist, post-intervention, and transfer to the operating room, similarly to the study carried out by Golden et al. [14]. VAS-P scores did not show significant differences between the groups until the third evaluation point, during the transfer to the gray area, at which time the control group exhibited lower anxiety scores (p<0.05). Although VAS provides parental perception, which is clinically important, they tend to be more subjective and can be influenced by parents' anxiety and expectations [16]. For this reason, it is recommended to use it in combination with more objective instruments like the mYPAS.

Regarding the mYPAS instrument, it is an observational checklist recognized as a reliable tool for assessing children's anxiety during the perioperative period. It consists of 22 items, with total scores ranging from 22.5 and 100, indicating different levels of anxiety [17]. In our study, mYPAS scores showed no significant difference between the study groups at the first evaluation. However, the number of patients who met the threshold for being considered anxious (>30 points) was significantly lower in the play therapy group. During the post-intervention evaluation, there were significant differences in the anxiety scores and in the number of anxious patients, both of which were lower in the play therapy group compared to control group. At the final evaluation point, there were no significant differences between the groups. However, the number of anxious patients was lower in the group that played and was transported in the toy car.

Our results were consistent with those found in the study by Liu et al., who observed that children who were transported in a toy car exhibited significantly lower levels of anxiety from the moment they got into the car until they entered the operating room, compared to other groups [15].

However, this study has several limitations. First, our sample size was small and limited to hernioplasties, without considering other elective surgeries. Second, toy car transportation was the only play-based intervention implemented, and the fact that it was not a structured play activity compromises the reproducibility of the intervention. Third, it would have been more appropriate to have an external evaluator blinded to the study, when determining the levels of anxiety with both scales. Fourth, the study design did not evaluate the post-anesthetic implications of the intervention, which would be a very interesting perspective. Finally, although randomization was adequately performed, a clinically relevant imbalance in baseline mYPAS scores was observed between groups. This imbalance may be related to the small sample size and the use of simple randomization, which may not ensure complete balance of baseline characteristics. This limitation could affect internal validity, as the control group may have been inherently more anxious. Therefore, results should be interpreted with caution. Additional studies are necessary to address these limitations and confirm the effectiveness of play therapy in reducing perioperative anxiety in children.

## Conclusions

The results of this study suggest that therapy involving play and transportation in a toy car is effective in preventing anxiety in pediatric patients undergoing inguinal hernioplasty. Among the strengths of the study are the use of a randomized controlled trial design, the use of a validated anxiety scale assessed by observers (mYPAS-SF), which was measured at multiple time points to track the trajectory of anxiety, and the comparison with an active control (midazolam), which has clinical relevance. Although there are other non-pharmacological strategies that reduce anxiety in the perioperative period, such as distraction techniques, tours of the operating room, as well as simulation, audiovisual, or music therapy techniques, we highlight free play therapy with a transport cart because it transforms the perioperative journey into an active and playful experience, not just a distraction strategy. In addition, it helps reduce anxiety from the loss of control to perceived threat, making it particularly effective in young children and in high-anxiety contexts. Therefore, we recommend these non-pharmacological anxiolytic strategies for pediatric patients in whom midazolam use may be challenging due to preparation, administration difficulties, supply shortages, allergies, or resistance to taking the medication.

## References

[1] Liu W, Xu R, Jia J, Shen Y, Li W, Bo L (2022). Research progress on risk factors of preoperative anxiety in children: a scoping review. Int J Environ Res Public Health.

[2] Kalk NJ, Nutt DJ, Lingford-Hughes AR (2011). The role of central noradrenergic dysregulation in anxiety disorders: evidence from clinical studies. J Psychopharmacol.

[3] Tafet GE, Nemeroff CB (2020). Pharmacological treatment of anxiety disorders: the role of the HPA axis. Front Psychiatry.

[4] Ariño-Braña P, Zareba MR, Ibáñez Montolio M, Visser M, Picó-Pérez M (2025). Influence of the HPA axis on anxiety-related processes: an RDoC overview considering their neural correlates. Curr Psychiatry Rep.

[5] Cameron OG, Abelson JL, Young EA (2004). Anxious and depressive disorders and their comorbidity: effect on central nervous system noradrenergic function. Biol Psychiatry.

[6] Crocq MA (2017). The history of generalized anxiety disorder as a diagnostic category. Dialogues Clin Neurosci.

[7] Drasković B, Simin JM, Kvrgić IM (2015). Psychological aspects of pediatric anesthesia. Med Pregl.

[8] Cai Y, Lopata L, Dodhia S, Monteleone M, Haddad J Jr, Sun LS (2018). Differences in postoperative maladaptive behavioral changes between partial and total tonsillectomy patients. Int J Pediatr Otorhinolaryngol.

[9] He HG, Zhu L, Chan SW, Klainin-Yobas P, Wang W (2015). The effectiveness of therapeutic play intervention in reducing perioperative anxiety, negative behaviors, and postoperative pain in children undergoing elective surgery: a systematic review. Pain Manag Nurs.

[10] Godino-Iáñez MJ, Martos-Cabrera MB, Suleiman-Martos N, Gómez-Urquiza JL, Vargas-Román K, Membrive-Jiménez MJ, Albendín-García L (2020). Play therapy as an intervention in hospitalized children: a systematic review. Healthcare (Basel).

[11] Jenkins BN, Fortier MA, Kaplan SH, Mayes LC, Kain ZN (2014). Development of a short version of the modified Yale Preoperative Anxiety Scale. Anesth Analg.

[12] Schulz KF, Altman DG, Moher D; CONSORT Group (2010). CONSORT 2010 statement: updated guidelines for reporting parallel group randomised trials. BMJ.

[13] Berghmans JM, Poley MJ, van der Ende J (2017). A Visual Analog Scale to assess anxiety in children during anesthesia induction (VAS-I): results supporting its validity in a sample of day care surgery patients. Paediatr Anaesth.

[14] Golden L, Pagala M, Sukhavasi S, Nagpal D, Ahmad A, Mahanta A (2006). Giving toys to children reduces their anxiety about receiving premedication for surgery. Anesth Analg.

[15] Liu PP, Sun Y, Wu C (2018). The effectiveness of transport in a toy car for reducing preoperative anxiety in preschool children: a randomised controlled prospective trial. Br J Anaesth.

[16] National Clinical Guideline Centre (UK) (2010). Sedation in Children and Young People: Sedation for Diagnostic and Therapeutic Procedures in Children and Young People. https://www.ncbi.nlm.nih.gov/books/NBK82227/.

[17] Kain ZN, Mayes LC, Cicchetti DV, Bagnall AL, Finley JD, Hofstadter MB (1997). The Yale Preoperative Anxiety Scale: how does it compare with a "gold standard"?. Anesth Analg.

